# The histone variant H3.3 G34W substitution in giant cell tumor of the bone link chromatin and RNA processing

**DOI:** 10.1038/s41598-017-13887-y

**Published:** 2017-10-18

**Authors:** Jinyeong Lim, Joo Hyun Park, Annika Baude, Yeongran Yoo, Yeon Kyu Lee, Christopher R. Schmidt, Jong Bae Park, Jörg Fellenberg, Josef Zustin, Florian Haller, Irene Krücken, Hyun Guy Kang, Yoon Jung Park, Christoph Plass, Anders M. Lindroth

**Affiliations:** 1 Graduate School of Cancer Science and Policy, Cancer Biomedical Science, National Cancer Center, Gyeonggi-do, Republic of Korea; 2 Division of Epigenomics and Cancer Risk Factors, German Cancer Research Center, Heidelberg, Germany; 3 Metabolism and Epigenetics Laboratory, Department of Nutritional Science and Food Management, Ewha Womans University, Seoul, Republic of Korea; 4 Research Center for Experimental Orthopedics, Clinic for Orthopedic and Trauma Surgery, University of Heidelberg, Heidelberg, Germany; 5 Department of Orthopaedics, University Medical Center Hamburg-Eppendorf, Hamburg, Germany; 6 Institute of Pathology, University Hospital Erlangen, Erlangen, Germany; 7 Institute of Pathology, University of Leipzig, Leipzig, Germany

## Abstract

While transcription as regulated by histones and their post-translational modifications has been well described, the function of histone variants in this process remains poorly characterized. Potentially important insight into this process pertain to the frequently occurring mutations of H3.3, leading to G34 substitutions in childhood glioblastoma and giant cell tumor of the bone (GCTB). In this study, we have established primary cell lines from GCTB patients and used them to uncover the influence of H3.3 G34W substitutions on cellular growth behavior, gene expression, and chromatin compaction. Primary cell lines with H3.3 G34W showed increased colony formation, infiltration and proliferation, known hallmarks of tumor development. Isogenic cell lines with H3.3 G34W recapitulated the increased proliferation observed in primary cells. Transcriptomic analysis of primary cells and tumor biopsies revealed slightly more downregulated gene expression, perhaps by increased chromatin compaction. We identified components related to splicing, most prominently hnRNPs, by immunoprecipitation and mass spectrometry that specifically interact with H3.3 G34W in the isogenic cell lines. RNA-sequencing analysis and hybridization-based validations further enforced splicing aberrations. Our data uncover a role for H3.3 in RNA processing and chromatin modulation that is blocked by the G34W substitution, potentially driving the tumorigenic process in GCTB.

## Introduction

Central to cancer progression is the deterioration of function and integrity of tumorigenic cells previously in a structured relationship with tissues and organs in the organism^1^. Function of viability is a relative term, but must in its simplest form convey to a strict and congruent program of order. Gain-of-function characteristics in cancer driver genes caused by genetic aberrations can readily overthrow this order. Histones, with their key and multifunctional properties, are central components of the cell particularly vulnerable to these forces^2^. When histones are mutated, they could retain critical functions in the nucleosome while simultaneously gain new and deleterious functions with direct influence on gene expression and chromatin integrity. It is therefore not surprising that mutations of the histones have been associated with cancer, but due to strong redundancy among canonical histones, they appear to be restricted to histone variants and slanted towards children and younger individuals^3^. Recurrent mutations in childhood glioblastoma have been reported, occurring in both histone variant H3.3 and H3.1^4,5^. Since there is large redundancy in genes encoding canonical histones, they are mainly dominant-negative mutations. The leading example is a mutation of H3.3 that produces K27M substitution (hereafter referred to as H3.3^K27M^) which sterically bind and block the function of the polycomb repressive complex 2^6–8^. This has dramatic consequences on the chromatin as lysine 27 trimethylation of histone H3 (H3K27me3) is drastically reduced, leading to transcriptomic and epigenomic aberrations genome wide that in turn drive a proliferative advantage on the course to cancer. Mutations in the very same gene have been identified in giant cell tumor of the bone (GCTB), although not in children but in younger adults^9^. Mutations of H3.3 in GCTB are almost exclusively leading to G34W substitutions (H3.3^G34W^), whereas in glioma they are G34R/V substitutions (H3.3^G34R/V^).

Why brain and bone are the only organs where H3.3 mutations appear to occur remain unknown. Detailed analysis of the normal function of H3.3 in mouse embryogenesis and differentiation have been performed by several laboratories^10^. The histone variant H3.3 becomes incorporated into the nucleosomes to facilitate euchromatinization and transcription^11^, but heterochromatic or repressed regions are also known targets^12^. H3.3 is involved in a diverse array of nuclear activities; among them nucleosome turnover, transcriptional activity, genome integrity, and replication^13–16^. To specifically address the role of H3.3 in cancer, we focused on bone tumors with H3.3 mutations.

Giant cell tumor of the bone is a locally aggressive but only rarely metastasizing benign neoplasm of the bone, occurring most frequently at the meta-epiphyseal regions of the long bones, that manifest itself as osteolytic lesions with significant bone destruction^17^. While the histological properties of the tumor have been well documented, the cytogenetics at base resolution has only recently been addressed, aided by the technological revolution of the high-throughput DNA sequencing methodology^9^. Surprisingly, recurrent mutations occur exclusively at the H3.3 locus H3F3A (leading to the H3.3^G34W^), suggesting that this most certainly is the sole contributor to the tumorigenic processes of GCTB.

Here we have analyzed the transcriptome and H3.3 interactome of GCTB by means of primary and isogenic cell lines harboring H3.3^G34W^ at the H3F3A locus. Several lines of evidence point towards a direct influence on the pre-mRNA processing as H3.3^G34W^ interacts with several components of the spliceosome. In addition, GCTB with H3.3^G34W^ poses a proliferative and infiltrative advantage that uncover important aspects of H3.3 in cancer and opens possibilities for new stratification regiments and treatments.

## Results

### GCTB samples with H3.3^G34W^ grow faster with infiltrative capacity

**Figure 1:**
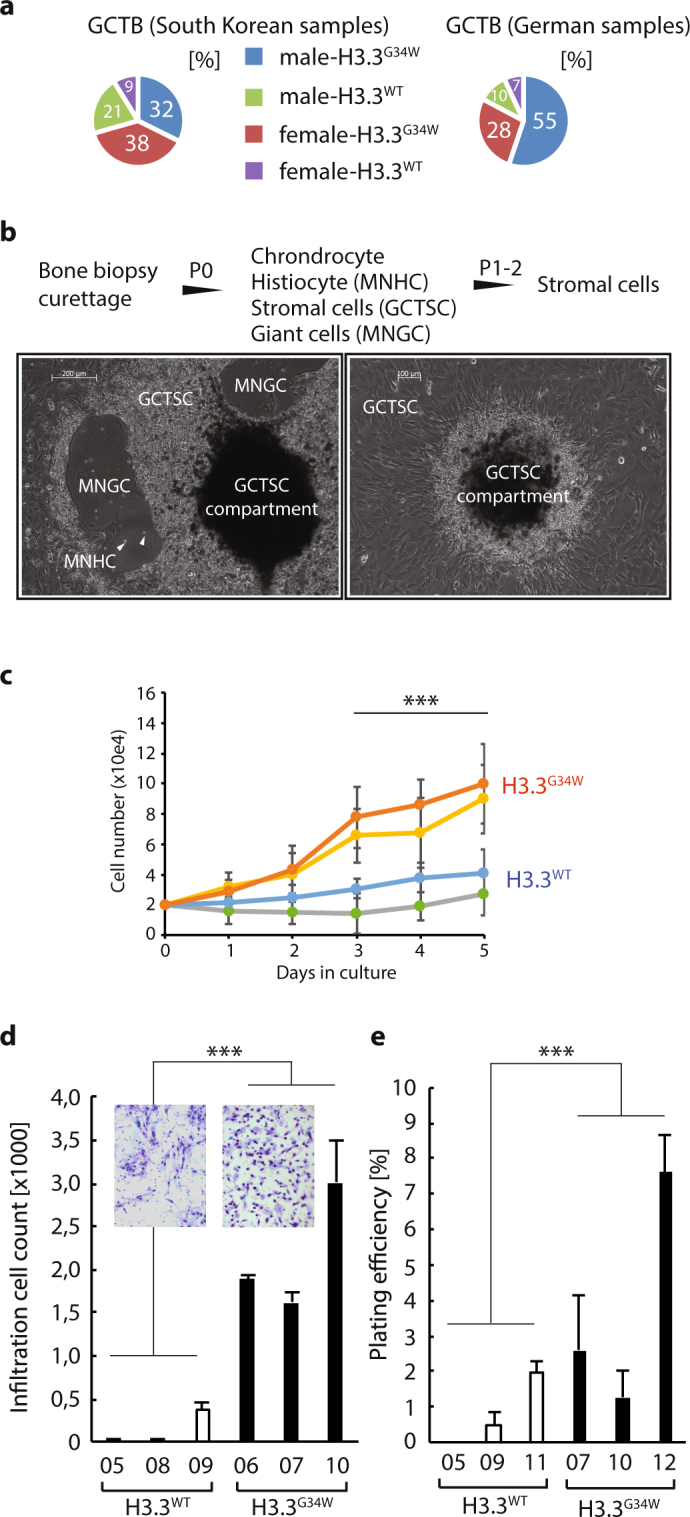
Giant cell tumor of the bone biopsies, from which primary cell lines display elevated growth, infiltration, and colony formation. (**a**) Pie charts outlining the distribution of the South Korean (left) and German (right) samples in this study, with or without H3.3^G34W^ divided by gender. Abbreviation: wild type H3.3, H3.3^WT^; H3.3 G34W substitution, H3.3^G34W^. (**b**) Procedure to establish primary cell lines from the GCTB biopsies. Pictures depict original culture (left) and enrichment of stromal cells (right) that typically emanate from a stromal cell compartment. Abbreviations: GCTSC, giant cell tumor stroma cells; MNHC, mononuclear histiocytic cells; multinucleated giant cells (MNGC). (**c**) Proliferative activity in H3.3^G34W^ compared to unmutated H3.3^WT^ primary cell lines as measured by automated cell counting after trypsinization (n = 4 for each value point, *P*-value < 0.001). (**d**) Boyden cell migration and infiltration assay measuring the primary GCTB cells ability to migrate towards a serum gradient (n = 4 for each value point, *P*-value < 0.001). (**e**) Colony formation assay where cells were seeded sparsely to allow colony formation and then stained with crystal violet for visualization and counting (n = 3 for each cell line, *P*-value < 0.001).

We have performed detailed analysis of 86 GCTB samples composed of a German and a South Korean cohort. We genotyped all samples for H3.3 mutations at the H3F3A locus to establish mutated versus unmutated groups for downstream analysis (Fig. 1a). We found H3.3^G34W^ at the following rate: 71% in the Korean and 83% in the German cohorts. The gender distribution of H3.3^G34W^ in the Korean samples were 46% males and 54% females, whilst in the German samples there were 67% males and 33% females with H3.3^G34W^ (Fig. 1a).

Since stratification of GCTB samples based on mutational status remains uncharted, we wanted to determine if there is a difference between locations of tumor and mutational incidence. We found that the distal femur and proximal tibia of the lower long bones are the areas where GCTB are the most frequent, especially in the German cohort. In the Korean cohort, the scapula/clavicular is also a prone area of GCTB. In the most common tumor locations, H3.3^G34W^ are as frequent 81–91% when considering both cohorts together (Supplementary Fig. S1A and B). A chi-square analysis comparing upper and lower long bones shows no significant difference between mutational status and location of the tumor (*P*-value 0.357).

While 72 of the 86 samples were fresh frozen or FFPE fixed material (Supplementary Table S1), 20 clinical biopsies (11 Korean and 9 German samples) were also obtained from patients undergoing surgical curettage of neoplastic tissue from which we established primary cell cultures (Fig. 1b). To enrich for the stromal compartment of the tumor, which is the only cell type carrying the H3.3^G34W^ substitution^9^, we disintegrated the tissue and cultured it under standard tissue culture conditions. We performed giant cell-specific TRAP staining^18^ on fixed and sectioned GCTB biopsies (Supplementary Fig. S1C). However, based on visual examination of the slides, we were not able to determine any obvious difference between H3.3^WT^ and H3.3^G34W^ samples (Supplementary Fig. S1C).

The procedure we used to enrich for the stromal compartment is briefly outlined in Fig. 1b. After two passages using this procedure, the stromal compartment was essentially devoid of other cells (e.g histiocytes and giant cells) and could be cultured separately (Fig. 1b). Interestingly, biopsies that were kept in culture in the original plate were spawning stromal cells for many months from enclosed compartments (Fig. 1b). When plates were trypsinized to remove stromal cells, the compartments remained attached and new stromal cells were growing out from them to eventually make the plates confluent again in a few days.

In previous reports on GCTB, a considerable difference in growth characteristics has been noted between cell lines^19^. Since this could be attributed to H3.3 mutational status, we tested proliferation, infiltration, and colony formation. We find that proliferation is increased in H3.3^G34W^ samples as compared to H3.3^WT^ (Fig. 1c). A key component of tumor growth is migration and infiltration, which we tested in Boyden assays. We found that infiltrative capacity is significantly elevated in H3.3^G34W^ over H3.3^WT^ (Fig. 1d). Cancer cells with stem cell properties tend to form colonies in culture, which is enhanced in H3.3^G34W^ versus H3.3^WT^ (Fig. 1e). These observations collectively suggest that H3.3^G34W^ take on more aggressive growth properties and potentially have a different pathogenesis than H3.3^WT^.

### Distinct gene expression profile of GCTB cells with H3.3^G34W^ potentially driving pathogenesis

**Figure 2:**
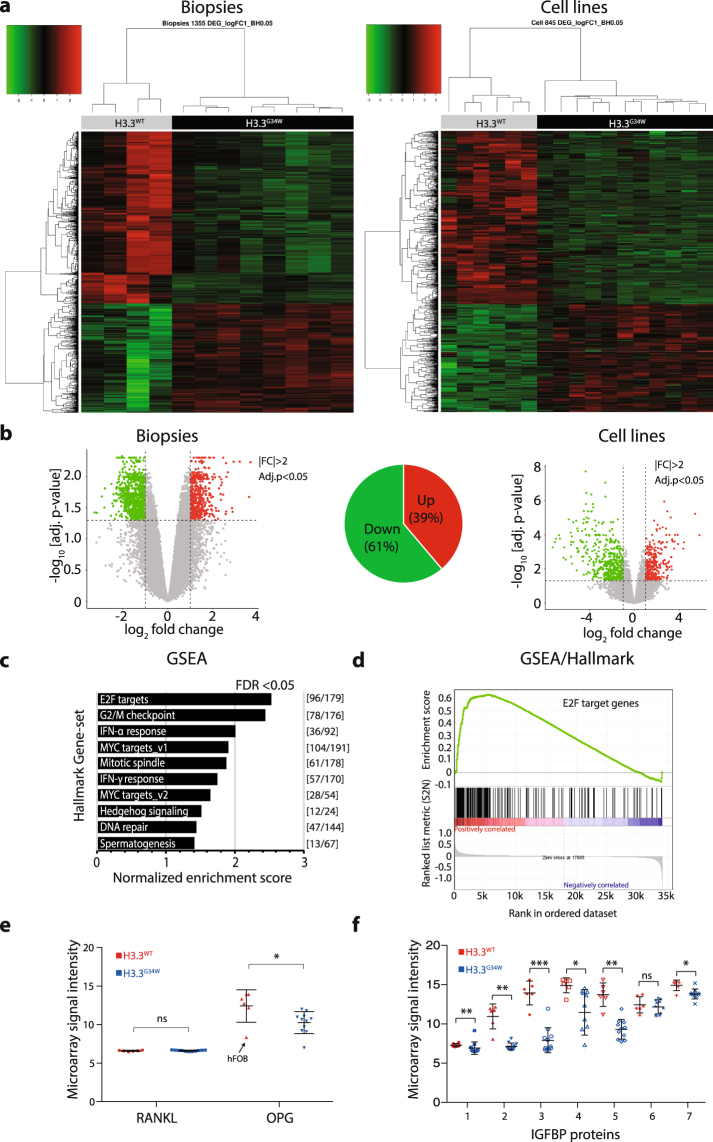
Distinct gene expression profiles of GCTB biopsies and primary cells targeted by E2F transcription factors. (**a**) Microarray gene expression profiles of significantly differentially expressed genes (DEG) displayed by supervised hierarchical clustering heatmaps and dendrogram of twelve biopsies and seventeen cell lines (Benjamini-Hochberg (BH) procedure, adj. *P*-value ≤0.05, |FC| ≥2). Number of DEGs in the heatmap were 1355 for the biopsies and 845 for the cell lines. Green is down and red is up-regulated DEGs. (**b**) Volcano plots of microarray expression data of biopsies (left) and primary cell lines (right). Thresholds calculated by BH procedure (FDR 0.05) and indicated with dashed lines (upregulated genes indicated with red dots and downregulated genes with green dots). Pie chart shows that 61% of DEGs are downregulated. |FC| ≥ 2 and −log_10_ adjusted *P*-values < 0.05. (**c**) Gene set enrichment analysis (GSEA ver. 2.2.2) of microarray data of biopsies and primary cell lines indicating significant Hallmark gene sets (FDR < 0.05). The number of differentially expressed genes for each hallmark category is indicated in the right margin. with E2F target category with the highest normalized enrichment score. (**d**) Enrichment plot indicating strong positive correlation (red) between differentially expressed genes and E2F target genes in the GSEA/Hallmark category. Negatively correlated genes (blue) are consequently less enriched. (**e**) Gene expression of the tumor-related RANKL (TNFSF11) and OPG (TNFRSF11B), where the RANKL-stabilizing OPG is downregulated while RANKL is not. One outlier data point, the osteoblast hFOB1.19 cell line, is marked with an arrow, suggesting a cell type specific discrepancy in gene expression. (**f**) Plot of the gene expression microarray data of the IGFBP gene family indicates a collective downregulation of most members among the H3.3^G34W^ tumor samples. The IGFBP gene family is also targeted by E2F transcription factors (Supplementary Fig. S3B).

To begin unraveling the cause behind the aggressive growth characteristics posed by H3.3^G34W^, we performed gene expression microarray analysis on both patient biopsies and established primary cell lines. We find that the divide in proliferative properties between H3.3^G34W^ and H3.3^WT^ samples also are reflected in the gene expression profiles, as they clearly separate in phylogenetic analysis (Fig. 2a). While we observe a relatively small number of genes to be differentially expressed (1355 in biopsies and 845 in cell lines, adjusted *P*-value < 0.05, |FC| ≥ 2), about 61% of them are downregulated in H3.3^G34W^ (Fig. 2b). As our strategy was to isolate and use the neoplastic stromal compartment for functional analysis, which also are the only cells carrying the H3.3^G34W^ substitution, we performed hierarchical clustering of biopsies and primary cell lines together. We find that the primary cell lines and biopsies cluster very well according to their mutational status (Supplementary Fig. S2). Like the separate analysis of biopsies and cell lines, 58% of the differentially expressed genes are downregulated when analyzed together (Supplementary Fig. S2B). The primary mesenchymal stromal cell line KM1234 cluster with H3.3^WT^ while the pre-osteoblast cell line hFOB1.19 cluster as an outgroup to both H3.3^WT^ and H3.3^G34W^ (Supplementary Fig. S2D).

We then tested for association to biological processes by performing gene set enrichment analysis (GSEA) of quintile-normalized microarray gene expression data. A large portion of genes converge on E2F target genes in the Hallmark GSEA gene set (Fig. 2c,d) and appear downregulated in H3.3^G34W^. We also find gene sets related to cell cycle-regulation as G2/M checkpoint and MYC target genes have been associated with these processes.

While GSEA showed a strong correlation with differentially expressed genes targeted by the E2F transcription factor family, we further looked at genes relevant for GCTB and proliferative properties with known E2F association. The tumor necrosis factor ligand superfamily member 11 (TNFSF11, or RANKL) has been shown to be highly expressed in GCTB stromal cells^20^, and to be the master regulator of osteoclast differentiation^21^. Its activity is inhibited by osteoprotegerin (TNFRSF11B, or OPG) which is a secreted decoy receptor of RANKL as a physiological inhibitor of RANKL-induced osteoclast formation^22^. While *RANKL* expression remains unchanged, we find that H3.3^G34W^ significantly downregulates *OPG*, potentially elevating the osteoclast activity of RANKL (Fig. 2e). This is supported by targeting of E2F with dimerizing protein, E2F/DP^23^, at the transcriptional start site of OPG (Supplementary Fig. S3A).

IGFBP proteins have known growth-inhibitory effects and inhibit proliferation by blocking insulin growth factors IGF1 and 2 to activate their receptor and stimulate proliferation^24^. The fourth member of the IGFBP gene family, IGFBP4, was previously shown to be silenced by DNA hypermethylation and associated with clonogenicity in GCTB^18^. In our expression data, we find that several members of the *IGFBP* family are significantly downregulated (Fig. 2f) and targeted by E2F/DP (Supplementary Fig. S3B), again suggesting that this transcription factor complex is dysregulated under H3.3^G34W^.

Here we have highlighted one relevant gene-pair and one gene family as examples of expression patterns in line with the aggressive pathogenesis of GCTB carrying the H3.3^G34W^ and thus, E2F deregulation could be major contributor to the tumorigenic process leading to overgrowth and bone destruction.

### Immunoprecipitations coupled with mass spectrometry uncover novel and common interacting proteins to H3.3^G34W^

Since the histone tails are preeminent binding sites for many chromatin remodelers, particularly to the numerous post-translational modifications, we investigated if the substitutions of H3.3 serve as binding sites for *novel* protein interactors and if *common* interactors will gain or lose stoichiometric binding as compared to unmutated H3.3.

**Figure 3:**
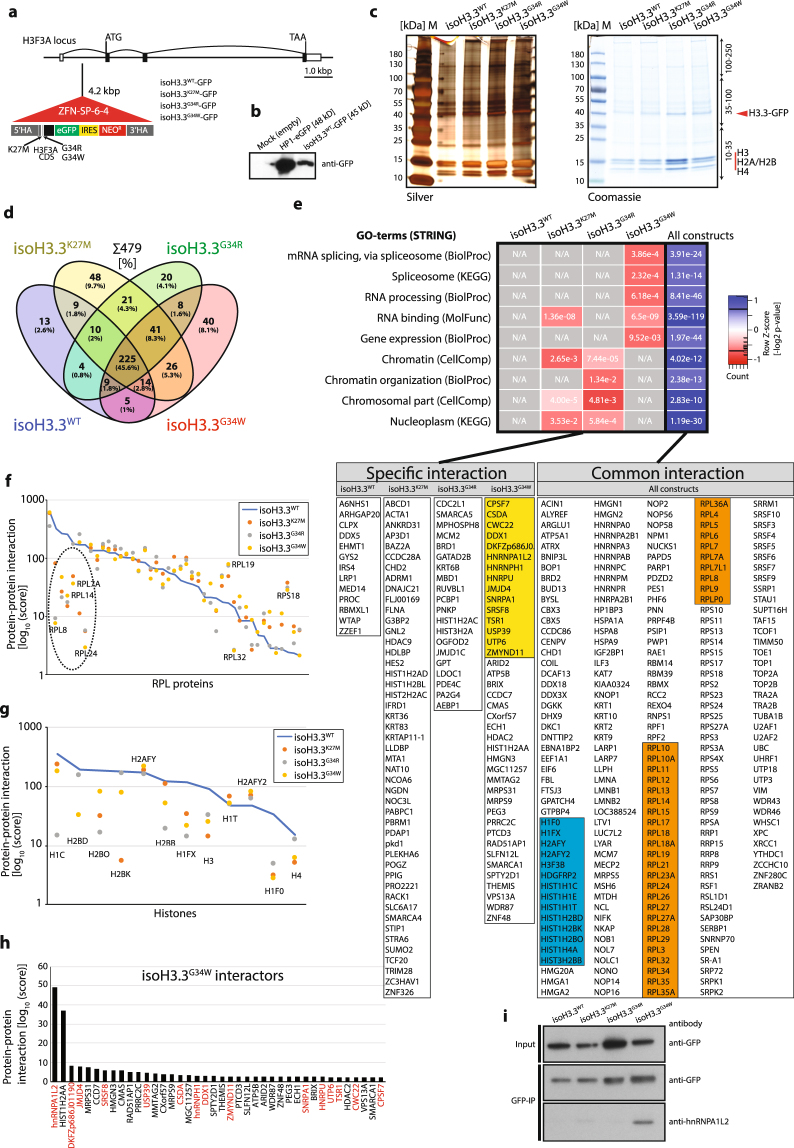
Immunoprecipitations coupled to mass spectrometry analysis of mutated and unmutated H3.3 reveal novel and common interacting proteins. (**a**) Illustration of H3.3 constructs targeting intron 1 of the endogenous H3F3A locus. The H3F3A CDS was fused to eGFP (with stop codon) and an IRES-separated Neo for antibiotic resistance. The IRES between GFP and Neo facilitates production of separate transcripts. The construct is driven by the endogenous H3F3A promoter (exon 1) and the downstream endogenous exon 2, 3 and 4 does not contain any mutations and is not translated. (**b**) Western blot verification of the H3.3^WT^ construct for GFP expression. Heterochromatin Protein1 (HP1)-GFP was used as positive control and mock empty vector as negative control. (**c**) PAGE-gel of immunoprecipitated protein lysates by anti-GFP antibody of the isogenic cell lines. The gels were stained with silver (left panel) and Coomassie (right panel). Each lane was divided into three equal slices before in-gel digestion and LC-MS/MS mass spectrometry analysis. The H3.3-GFP band is indicated, and associated histones. (**d**) Venn diagram indicating relationship between constructs and immunoprecipitated proteins, identified by LC-MS/MS. Numbers are individual proteins and in parentheses percentage of total. (**e**) GO-term analysis of interacting proteins using the STRING application (string-db.org). Heatmap of GO-terms based on z-score transformed [−log_2_] *P*-values. Individual proteins identified are listed below the heatmap, divided in columns. Colored boxes represent spliceosome-associated proteins (yellow), RPL proteins (orange) and histones (blue). Common interactions are proteins bound to all isoH3.3-lines. (**f**) RPL protein family members plotted as a function of protein-protein interaction [log_10_ score]. Dashed line circle indicates RPL proteins with highest scores in unmutated construct (blue line) with the biggest drop of scores. (**g**) Histones, especially H1 and H2B, show reduced protein-protein interaction score as compared to unmutated construct (blue line). (**h**) Bar graph with the H3.3^G34W^-interacting proteins, indicating hnRNPA1L2 with the highest score. Protein names in red indicate splicing-associated factors. (**i**) Western blot analysis of GFP-immunoprecipitations from total protein extracts of the isoH3.3 lines. Input indicates H3.3-GFP in total protein extracts and Co-IP the amount of H3.3-GFP precipitated after GFP-bead binding and elusion. The same filter as GFP-IP was used to detect hnRNPA1L2.

We first made a construct that carries a GFP epitope tag C-terminal to the H3.3 CDS of H3F3A, so that we could easily precipitate it with an epitope tag antibody (Fig. 3a). We then used this construct to make three additional constructs with mutations by site-directed mutagenesis, individually leading to K27M, G34R, and G34W substitutions, and WT as control. These constructs were then transfected into HEK293 cells along with specifically designed zinc finger nuclease targeting arms to facilitate locus specific recombination by nicking the DNA with the tethered TypeIIS enzyme *Fok*I, and to establish isogenic cell lines targeting the endogenous first intron of the H3F3A locus. They were hereafter named isoH3.3^WT^, isoH3.3^K27M^, isoH3.3^G34R^, or isoH3.3^G34W^, to indicate the specific substitutions and to distinguish them from the primary cell line annotations. The constructs were designed to be expressed by the endogenous H3F3A promoter. Established isogenic cell lines were confirmed with PCR, and with Southern blot analysis (Supplementary Fig. S4A), and we also confirmed that the construct expressed H3.3-GFP fusion protein as expected (Fig. 3b). We have been able to generate two to three individual clones per gene targeting construct (Supplementary Fig. S4A).

We seeded and harvested the same number of isogenic cells for each of the four constructs and performed co-immunoprecipitations with GFP Trap-A beads to pull down proteins interacting with the constructs and a parental, untransfected cell line. We confirmed equal loading between the lanes on an SDS-PAGE (Fig. 3c) and an identical parallel gel was subjected to in-gel trypsin digestion and LC-MS/MS peptide separation.

A protein score was generated by the SEQUEST algorithm that considers peptide charge and the number of peptides for the interaction between H3.3-GFP and precipitated proteins. A complete list of interactors can be found in Supplementary Table S2. While our focus was on H3.3^G34W^, we used the other three constructs as means to understand common H3.3 interacting proteins and as a reference point for the specific H3.3^G34W^ interactors. The untagged parental cell line did not pull down any proteins that we could analyze.

About 45% of all identified proteins (Protein [score] >2) were common interactors (Fig. 3d,e), including the known chaperone ATRX that incorporates H3.3 at heterochromatic sites^25^. The isoH3.3^WT^ cell line had the lowest number of specific interactors with its 2.6%, while isoH3.3^K27M^ and isoH3.3^G34W^ had the highest, 9.7% and 8.1%, respectively. We divided all identified proteins into *Specific* or *Common interaction* proteins to the constructs, where *Specific* refer to proteins only interacting with one construct, and where *Common* refer to proteins that interact with all constructs but often with different affinity depending on mutation. When performing GO-term analysis (STRING), we find that isoH3.3^G34W^ interacts with factors associated with the spliceosome, RNA processing, ribosome biogenesis, and gene expression (Fig. 3e). We did not see a correlation between gene expression and loss of interaction as a potential explanation for the observed reduction of interaction (Supplementary Fig. S5A).

Two protein families in the *Common interaction* category stood out as being of special interest: The Like ribosomal protein (RPL) family (Fig. 3f), and Histones (Fig. 3g). The strongest binding RPLs (e.g RPL7A, RPL8, and RPL24) lost most of their interaction with mutated H3.3 as compared to unmutated H3.3, especially isoH3.3^G34W^ (Fig. 3f). While the RPL proteins play an important role in ribosome assembly, ribosome-independent functions have also been documented^26^. RPLs are synthesized in the cytoplasm and rapidly imported into the nucleolus where they participate in the assembly of the pre-ribosome rRNA. Ribosomal proteins are inherently unstable and will be degraded if they are not incorporated, ultimately leading to ribosomal stress^26^. Since RPLs can act both as oncogenes and tumor suppressors, their stoichiometric relationship appears to be important. Reduced binding of RPLs to H3.3 should lead to an increase of released protein, which have been shown in some studies to have oncogenic potential by inhibiting p53, activating NF-κb, or inducing cyclin D1 and D3^26^.

While H2AA appear to bind specifically to isoH3.3^G34W^ (Fig. 3h)., several other histones lose interaction with isoH3.3 mutations compared to isoH3.3^WT^, especially H2B and the linker histone H1F0 (Fig. 3g). It has been shown that H3.3 destabilize the nucleosome when incorporated, facilitating DNA access, but also antagonizing H1, blocking the linker histone from compacting the chromatin^27^.

Among isoH3.3^G34W^ interactors, we found that hnRNPA1L2 produced the highest score, while two other hnRNP family members, hnRNPH1 and hnRNPU, also interacted but with lower scores (Fig. 3h). We validated the interaction to isoH3.3^G34W^ with an independent co-IP of the constructs in a Western blot analysis with a specific antibody to hnRNPA1L2, indicating that isoH3.3^G34W^ specifically bound hnRNPA1L2 (Fig. 3i). The hnRNPs are *trans*-acting splice factors that regulate alternative splicing as both repressors and activators in a sequence-context dependent manner^28^. Other spliceosome-associated factors such as DKFZp686J01190 (homologous to DDX5), JMJD4, and SRSF8 were among interactors with relatively high score. The protein with the second highest score was histone H2A family member a (H2AA), which suggests it forms a tight interaction that potentially affects chromatin compaction.

### Chromatin compaction defects in H3.3^G34W^

**Figure 4:**
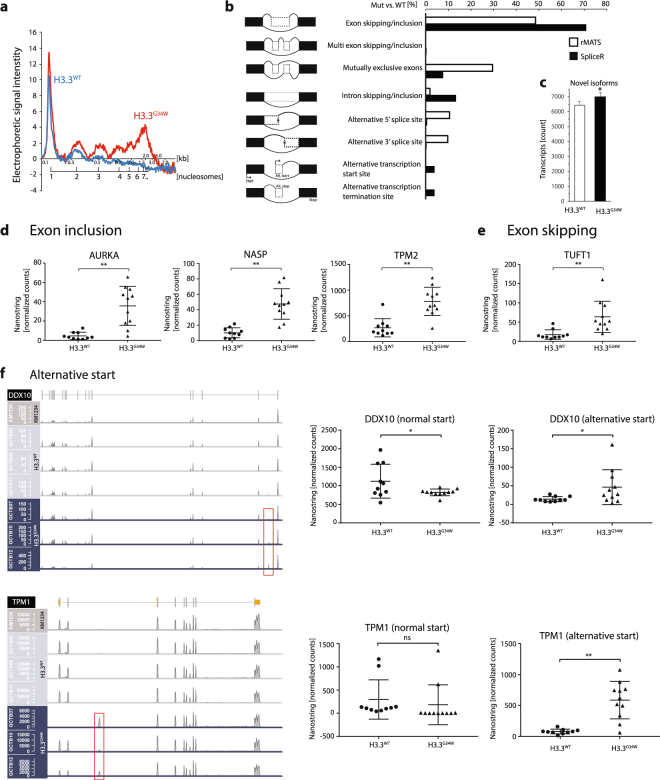
RNA-sequencing analysis of three H3.3^WT^ and three H3.3^G34W^ primary cell lines show distinct splicing aberrations. (**a**) Electropherogram of MNase assay estimating chromatin compaction of mutated and unmutated primary cells. Plot based on 60 min incubation with MNase of H3.3^WT^ (blue line) and H3.3^G34W^ (red line) primary cell lines and BioAnalyzer data collection. (**b**) RNA splicing events of H3.3^G34W^ vs. H3.3^WT^ primary cells, extracted by the rMATS and SpliceR algorithms. The increased splicing events in H3.3^G34W^ when compared to H3.3^WT^ are given in [%]. (**c**) Plot of the significant increase of potentially novel isoforms. The data is based on the Cufflinks category *j* that calls for potentially novel isoforms (fragments) with at least one splice junction shared with a reference transcript. (**d**) Three examples (AURKA, NASP, and TPM2) of exon inclusion events in H3.3^G34W^, first observed in RNA-seq. and here validated with the nCounter RNA-based hybridization technology. (**e**) Exon skipping in TUFT1 occur in H3.3^G34W^, and probe detects junction of flanking exons to the skipped exon. (**f**) Alternative transcriptional start sites at the *DDX10* and *TPM1* locus. Increased read counts in their first or second introns indicated by red boxes. Plot of nCounter validations are shown in the right panel, where loss of normal start correlates with gain of alternative start.

Motivated by observations that mutations of H3.3 affect heterochromatin formation^27,29,30^, we wanted to investigate if H3.3^G34W^ also caused changes to chromatin compaction. We therefore performed micrococcal nuclease assays (MNase assay) of primary cell lines with H3.3^WT^ or H3.3^G34W^, which estimates global changes to the chromatin. MNase cuts the DNA in the linker region between nucleosomes (nucleosome plus linker region is about 200 bp), and a compact chromatin is poorly digested with MNase. We find that chromatin is more compact in H3.3^G34W^ since it is more resistant to digestion with MNase compared to H3.3^WT^ (Fig. 4a). This is in line with the observation that isoH3.3^G34W^ lose interaction with H1 (Fig. 3g), potentially allowing H1 to facilitate chromatin compaction, and while simultaneously sequester H2A (Fig. 3h). All this could collectively contribute to the reduced gene expression that we observe in our transcriptome analysis (Fig. 2b).

### Detailed transcriptome analysis reveal RNA processing aberrations

Both microarray analysis (elevated splice form probe count, data not shown) and mass spectrometry data suggested that H3.3^G34W^ cause aberrations to the RNA processing machinery. To gain detailed insight into gene specific splicing, we performed RNA sequencing of three H3.3^WT^ and three H3.3^G34W^ primary GCTB cell lines. Using two different Linux-based softwares, rMATS and SpliceR, we found that *exon skipping/inclusion* were the most common aberration when comparing the two groups, additionally extending into the *mutually exclusive exons*-feature (Fig. 4b). Exon inclusion (1254 events observed) was twice as common as exon skipping (661 events observed). We also found that the number of alternative transcriptional start sites and termination sites increased, but to a lesser extent.

The number of transcripts annotated by the Cufflinks algorithm as potential novel isoform was also significantly elevated in H3.3^G34W^ (Fig. 4c).

We validated these findings by performing hybridization-based quantitative analysis of total RNA from 24 GCTB biopsies with the nCounter technology (Nanostring Tech.). We found several transcripts with exon inclusion, e.g. *AURKA*, *NASP*, and *TPM2* (Fig. 4d). AURKA, a chromosome segregation related kinase, is yet another example of the danger of an overexpressed kinase in cancer. NASP is a known histone H1 chaperone contributing to chromatin compaction, but also to cell proliferation in cancer cells and histone import to the nucleus^31^. While we could validate several transcripts with exon inclusion, we found relatively few transcripts with exon skipping. *TUFT1* is the best example, related to mesenchymal stem cell functions. Additional examples that we validated were alternative start sites for *FLRT2* and *UNG*, and exon inclusion events in *LGALS8* and exon skipping in *ZDHHC7* (Supplementary Fig. S6). Whether the observed *exon skipping/inclusion* feature results in aberrant transcripts, altered transcript stability, or extended open reading frames, remains to be investigated.

The SpliceR algorithm also indicated that there is an increase of alternative transcriptional start sites, e.g. *DDX10* and *TPM1* (Fig. 4f). TPM1 is a well described actin-binding protein essential for muscle contraction and cytoskeleton, while DDX10 is a DEAD-box containing RNA helicase with functions in ribosome and spliceosome assembly.

Together these results show that H3.3^G34W^ significantly alter the reading frame of a subset of transcripts that are important in chromatin function, RNA metabolism, and cell proliferation.

### H3.3^G34W^ expression in the osteosarcoma cell line MG63 shows increased proliferation

The biological significance of the proliferative advantage observed in the primary cell lines with H3.3^G34W^ would be strengthen if they were recapitulated in a relevant cell line where we have introduced H3.3^G34W^. We therefore established isogenic cell lines in the bone tumor-related osteosarcoma cell line MG63, with the H3.3^WT^ and H3.3^G34W^ targeting constructs, as was described for the HEK293 cell lines. The lines were named MG63-isoH3.3^WT^ and MG63-isoH3.3^G34W^, and were confirmed with PCR, Southern blot analysis and GFP expression similar to the isogenic HEK293 cells (Supplementary Fig. S4B and C).

**Figure 5:**
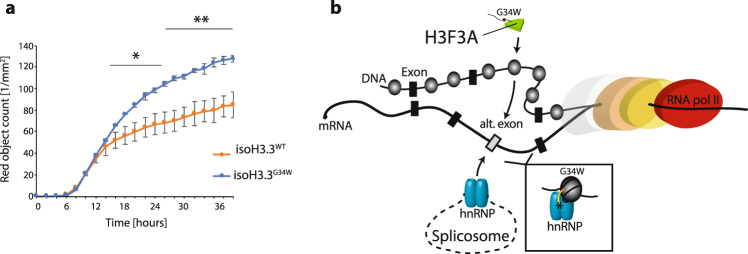
Isogenic H3.3^G34W^ cell line recapitulates elevated growth observed in primary GCTB cells. (**a**) Plot of proliferation rate of isogenic MG63 cells with H3.3^WT^ or H3.3^G34W^. Student’s t-test, *P*-value < 0.05 (*), *P*-value < 0.01 (**). (**b**) Model for the placement of H3.3^G34W^ in the splicing mechanism.

The isogenic cell line MG63-isoH3.3^G34W^ proliferated with significantly increased rate compared to MG63-isoH3.3^WT^ (Fig. 5a). This perfectly recapitulates the growth advantage posed by H3.3^G34W^ that we observed in the established primary GCTB cell lines, indicating that H3.3 is fundamentally important in controlling cell proliferation.

## Discussion

Histone variants in the human genome, currently numbered at a dozen and a half, has been shown to be of crucial importance in various cellular processes related to development and disease^32^. Histone variant H3.3 is no exception and several reports have shown H3.3 to be involved in both processes. We have therefore characterized the influence of the highly recurrent mutations of the N-terminal tail, with special emphasis on the G34W substitution. Until now, two tumor entities have been shown to carry G34 substitutions: glioblastoma and giant cell tumor of the bone. In both cases, the incidence is particularly slanted towards younger individuals, although it is less pronounced in the bone. Part of the explanation appears to be that H3.3 play a critical role in developmental processes^27^. Furthermore, as early as at the gamete stage, H3.3 is present in the oocyte and then appears active throughout the early embryo^33^. Transcriptionally active chromatin is well correlated with histone methylations of the histone 3 class, particularly at lysine 4, 36, and 79, and general acetylation of other lysines^11^, while transcriptionally silent chromatin correlates with lysine and 27^34^. Mutations of H3.3 have been shown to affect epigenetic patterning, but how that influences formation of tumors remains elusive.

We collected samples from patients with GCTB to gain insight into the tumorigenic processes driven by H3.3^G34W^ substitutions. We retrieved tumor samples from Germany and South Korea, and compared the two geographically separated cohorts. While several morphologic mimics of GCTB exist, e.g chondroblastoma, aneurysmal bone cysts, fibrous metaphyseal defects and giant-cell rich variants of low-grade osteosarcoma, all samples of the current study fulfilled morphologic and radiologic criteria of GCTB. While mutation incidence of the most common areas of GCTB in our cohorts ranged from 81–91% (Supplementary Fig. S1A and B), the incidence of H3.3 mutations in GCTB have been reported to be as high as 92% in another study^9^.

The proliferative activity of stromal cells was elevated in H3.3^G34W^, which remained in later passages (Supplementary Fig. S1D–E). However, cells undergo gradual senescence after about 10 passages whereupon they enlarge and flatten out, develop filopodia, and stop dividing.

Most downregulated genes in H3.3^G34W^ are E2F targets (Fig. 2c,d and Supplementary Table S2), which suggest that the high proliferative activity carried by the H3.3^G34W^ cells may be a result of silencing of critical negative regulators of the cell cycle such as the E2Fs. When specifically examining GCTB relevant genes targeted by the E2F transcription factors, we found that RANKL and OPG are differentially targeted (Supplementary Fig. S3A). OPG gene expression is downregulated, and its transcription start site is targeted by E2F/DP. In contrast, RANKL lack E2F association at the TSS. The IGFBP family of genes are also common targets of E2F (Supplementary Fig. S3B). Together, this indicates that H3.3 cooperates with E2F transcription factors and the H3.3^G34W^ leads to loss of E2F control and mostly downregulation of their canonical targets.

We and others previously reported that K27M substitutions of H3.3 sterically blocked the catalytic activity of the polycomb repressive complex 2 (PRC2), resulting in rapid H3K27me3 loss genome-wide^6–8^. We argued that other recurrent substitutions of the H3.3 N-terminal tail would serve as binding site for novel, yet unknown proteins that could explain both the normal function of H3.3 as well as their dominant-negative features. We approached this experimentally by engineering an epitope-tagged H3.3, targeting the endogenous locus, to facilitate GFP-purification of novel interacting proteins. We made a panel of four H3.3 substitutions; unmutated WT, K27M, G34R, and G34W, resulting in isoH3.3^WT^, isoH3.3^K27M^, isoH3.3^G34R^, and isoH3.3^G34W^. Using these constructs, we uncovered novel interactors and highlighted previously described interactors, substantially contributing to our understanding of H3.3 function. It has been suggested that H3.3 is involved in facilitating an open chromatin configuration, since it is incorporated into the male pronucleus after fertilization, contributes to the depletion of heterochromatin protein 1 (HP1), acquires histone modifications associated with active transcription, and blocks linker histone H1 from chromatin compaction^13,34^. Our native immunoprecipitations and mass spectrometry analysis of isoH3.3^G34W^ provide further insights, since histone H1 binds less efficient to mutated H3.3, including the G34W (Fig. 3g), and we do observe a more condensed chromatin (Fig. 4a). Since knockdown of H3.3 with morpholinos in mouse embryonic stem cells leads to a condensation of chromatin by lack of counterbalancing linker histone H1, we sought to determine if the H3.3^G34W^ affect condensation in a similar manner^27^. Our micrococcal nuclease assays suggest that the chromatin is more compact in H3.3^G34W^ compared to H3.3^WT^, suggesting that blocking H1 is no longer effective (Fig. 4a, Supplementary Fig. S5B,C).

While the unmutated isoH3.3^WT^ pulled down very few novel proteins, the isoH3.3^G34W^ and isoH3.3^K27M^ were particularly prone binding sites, more than three times the number of isoH3.3^WT^ (Fig. 3d). The most pronounced category of proteins that isoH3.3^G34W^ interacts with, according to our GO term analysis, are components of the spliceosome and RNA processing machinery (Fig. 3e). This is especially significant with heterogeneous nuclear ribonucleoprotein A1L2 (hnRNPA1L2) that is the strongest specific interactor to isoH3.3^G34W^ (Fig. 3h). Two other members of the hnRNP family interact with isoH3.3^G34W^, hnRNPH1 and hnRNPU. Other interacting splice-factors are JMJD4, SRSF8, and USP39 (a.k.a. hSAD). Together, the association with these factors suggest close links to the spliceosome and pre-mRNA processing machinery. However, further analysis is required to establish how H3.3 and the spliceosome sub-complexes intersect in the processing of primary RNA transcripts.

Common interactors (i.e. shared by all constructs, including isoH3.3^WT^) pulled down the most number of proteins, resulting in about 45% of the total interactors (Fig. 3e). We find that they represent all GO-terms presented by the individual mutations, suggesting that additional proteins participate in GO categories other than the specifically interacting ones. We reason that the specific interactors provide a functional discrepancy that contributes to the observed difference in proliferation, gene expression and splicing. The single largest group of common interactors are represented by the ribosomal proteins RPL and RPS (Fig. 3f). Ribosomal proteins are enriched in the nucleolus to participate in the assembly of pre-ribosomes^26^. Remarkably, H3.3 interacts with the RPL sub-family of proteins, containing some 50 odd members, and the association of some members is weakened by mutations of its N-terminal tail. If this leads to a reduced assembly or number of ribosomes remains to be investigated, but it likely put constrains on differentiation as has been shown in mouse knockouts of RPL29 that leads to global skeletal growth defects^35^. If H3.3 participate in the assembly of ribosomes by linking ribosomal proteins remain to be shown, but newly synthesized and unassembled components of the ribosome are quickly degraded by the proteasome system in the nucleoplasm^36^. This could lead to a phenomenon known as *ribosomal stress* where ribosome biogenesis is impaired, eventually affecting translation^26^. This has previously been reported as contributing to cancer progression. Proteins interacting with the highest score to the isoH3.3^WT^ were also the ones that lost the most in interaction with H3.3 when mutated. We also found that histones, especially of the H2B and H1 categories but also H3 and H4, had markedly reduced interaction with the H3.3 substitutions (Fig. 3g). This was particularly striking for the linker and chromatin compaction histone H1, emphasizing the role H3.3 has in inhibiting H1’s compacting ability^32^.

Mechanistically, the mass spectrometry analysis clearly showed that the major interacting components to the isoH3.3^G34W^ were of the spliceosome machinery. We verified splicing defects by performing RNA-sequencing on primary cell lines, and found that the major aberration was exon skipping/inclusion (Fig. 4b). This could be created by a faulty processing by the spliceosome, linked to the debarred function of activating and repressing *trans*-acting proteins^37^, like hnRNPA1L2. The critical bridging between exonic splicing silencers and neighboring exons by hnRNPs is potentially lost by this interaction and may instead facilitate novel exon inclusion. This can then inhibit stable interaction to the U6 snRNA, leading to loss of spliceosomal catalysis where hnRNP regulation is critical^38^. The connections between hnRNPs, H3.3 mutations and splicing alterations remain to be resolved in detail in future studies.

To recapitulate the observed proliferative advantage that the primary cells display, we established isogenic cell lines carrying H3.3^G34W^ in the osteosarcoma cell line MG63. Remarkably, the cells proliferate with elevated vigor when carrying the mutated construct at the endogenous locus (Fig. 5a). We conclude that MG63-isoH3.3^G34W^ alone can trigger high proliferative activity, perhaps by interfering with the E2F transcription factor targeting of cell cycle regulating genes. However, the details of this interaction remain to be investigated.

In this study, we show that the H3.3 histone variant influences transcription, the chromatin structure, and interact with the RNA processing machinery. Functions that fail when it accumulates H3.3^G34W^ substitutions, leading to hyper-proliferative activity towards cancer in the bone (Fig. 5b). When these normally dynamic, and presumably critically orchestrated events fail to fold, they display aberrations that lead to disease and aberrant development. Recent estimates of somatic mutations in chromatin remodelers suggest that cancer are strongly influenced by those proteins^39^, which calls for the development of specific chromatin-related combinatorial drugs^40^. Our findings open for future studies to resolve the intimate relationship between chromatin, RNA processing and cellular proliferation in cancer.

## Methods

### Sample collection

We have analyzed a total of 88 samples (86 GCTBs and 2 control samples), of which 9 were from the Heidelberg University Hospital (Germany), 21 from Gemeinschaftspraxis Pathologie-Hamburg (Germany), 20 from the Institute of Pathology Leipzig (Germany), 36 from the National Cancer Center (Rep. of Korea) and 2 reference cell lines (Supplementary Table S1). In the evaluation of the preoperative clinical state of GCTB, the standard radiographic grading system^41^ and operation type criteria^42^ were used. German samples were formalin fixed paraffin embedded (FFPE), while the Korean samples were fresh frozen stored in N_2_(liq.). A total of 20 post-curettage biopsies from the two clinics were collected to establish primary cell lines. Informed consent to analyse tumour tissue and to publish clinical details was obtained from all individuals included in the study. The use of patient samples and the experiments performed in this study was approved by and in accordance with guidelines and regulations by the Ethics Committees of the University of Heidelberg, University of Leipzig, University Medical Center Hamburg-Eppendorf, and the National Cancer Center of Korea (IRB NCC2015-0070).

### Primary cell culturing conditions

To enrich for the stromal compartment of the GCTB, we minced tissues in culture media (DMEM, 10% fetal bovine serum and 1% penicillin/streptomycin (HyClone)), supplemented with 1.5 mg/mL collagenase B (Roche Diagnostics), prior to incubating 1 hour at 37 °C. The disintegrated tissues were collected and spun down, and the pellets were washed twice with PBS. Resuspended pellets were incubated in cell culture media and incubated for 24 hours in a 37 °C tissue culture incubator (Thermo Fisher Scientific, 5% CO_2,_ 80% humidity). Attached cells were treated with 0.05% trypsin-EDTA for 2 minutes to remove loosely attached cells, followed by 0.25% trypsin-EDTA for 5–8 minutes at 37 °C to remove the remaining cells (stromal compartment).

### Proliferation, infiltration and colony formation assays

#### GCTB stromal cell and isogenic cell lines proliferation assay

Proliferation of primary GCTB stromal cells and isogenic cell lines were measured post trypsinization by automated cell counting with the Cell Counter model R1 (Olympus).

Proliferation was also measured by the IncuCyte ZOOM System real-time instrumentation (Essen Bioscience). We seeded 1,000 cells in a 96-well cell culture dish. To stain nuclei, 4% (v/v) NucLight BacMam 3.0 Reagent (mKate2, red fluorescent protein) was added and the dish was incubated at ambient temperature for 20 minutes and then placed in the IncuCyte in a tissue culture incubator for 30 minutes prior to scanning. Cell images were captured every 2 hours (10x) and the scanned images were analyzed by IncuCyte ZOOM^TM^ 2015A software (Essen BioScience, USA) after 20 hours.

All measurements were done in duplicates and in two different experiments, and statistics performed with Student’s t-test.

#### Cell migration and infiltration assay

We measured the relative cell migration rate, or infiltration rate, by seeding early passage primary stromal cells in a Boyden chamber containing a micro-porous membrane, allowing cells to migrate chemotactically towards growth-factor containing media^43^. Each chamber cup, or Transwell Permeable Supports (Corning) fit a 24-well cell culture dish and in the bottom of the chamber Matrigel was added, which consists of a reconstituted basement membrane preparation (Corning). Matrigel was diluted 1:8 in ice-cold serum-free media (SFM), and 100 µl was added per cup and transferred to a well with 700 µl SFM, incubated overnight in a standard growth incubator for 24 hours. Media was removed from the cup, rinsed with SFM and 25,000 cells/100 µl were seeded, and transferred to a well with 10% FBS-containing DMEM media. After 15 hours, cups were removed and stained with the Diff Quik kit (Sysmex Asia Pacific). Membrane were removed, washed and mounted on a slide for counting in a phase-contrast microscope.

#### Cell colony formation assay

Early passage primary GCTB stromal cells were seeded sparsely at 500 cells/well in a 12-well cell culture dish^19^. After 14 days of culturing in standard media (10% FBS in DMEM), cells were fixed and stained with a solution containing 72% methanol, 3.7% formaldehyde and 0.25% crystal violet (Sigma) for 10 min in room temperature. Wells were washed with water and let dry. After counting, plating efficiency was calculated as 100 × (number of colonies)/(number of seeded cells).

### Establishment of isogenic cell lines

We generated independent knock-in cell lines with the zinc finger gene targeting technology, based on design of targeting recognition sites by the Zink finger Consortium web application^44^. Five targeting sites of the H3F3A locus were tested for effective targeting after design of targeting oligos and cloned into the two zinc finger subunits-encoded vectors pAC HA nIL2RGL hNeeai_1140 and _1141. Test-targeting were performed with the Surveyor mutation detection kit (Transgenomic). The targeting constructs containing the CDS of H3F3A were ligated into the pBluescript II (SK-) vector, together with an in-frame eGFP locus and IRES separating Neo-selection cassette. After establishing a wild type construct H3.3^WT^, site-directed mutagenesis (Agilent Technologies) was used to introduce mutations producing H3.3^K27M^, H3.3 ^G34R^, and H3.3^G34W^ substitutions. The constructs were used to target the endogenous H3F3A locus in HEK293 (kidney) cells using the Nucleofector-based system AMAXA (Lonza). After transfections, according to the AMAXA protocol, cells were passaged and kept on G418 antibiotic selection for at least 4 weeks before sorting for individual clones with GFP channeling in a FACSAria III flow cytometer (BD Biosciences). Transfection of the osteosarcoma cell line MG63, was done by electroporation of the exact same constructs with NEPA21 super electroporator (Nepa Gene Co.) according to the manufacturer’s instructions. The transfected MG63 cells were cultured in MEM and 10% FBS under standard culture conditions, and kept under G418 selection for 4 weeks before sorting as described above.

### Transcriptome analysis (microarray and RNA-seq)

We performed gene expression microarray analysis on 29 samples, of which 9 were German primary cell lines, 12 were Korean biopsies and 6 Korean cell lines, and 2 were control cell lines. Control cell lines were the bone-marrow derived mesenchymal stromal cell line KM1234 and the hFOB1.19 osteoblast cell line. Collectively, 19 samples were H3.3^G34W^ and 10 were H3.3^WT^. The samples were run on a HumanHT-12 v4 Expression BeadChip (Illumina).

RNA sequencing was performed on 6 Korean primary cell line samples of which 3 were H3.3^G34W^ and 3 were H3.3^WT^. We employed TrueSeq RNA kit for library preparation and paired-end sequencing on an Illumina HiSeq. 2500 instrument, and received on average 14 Gbp read bases per sample with a Q30 phred quality score of 91–96%.

### Immunoprecipitation, liquid chromatography and tandem mass spectrometry analysis (LC-MS/MS)

The GFP-expressing isogenic cell lines were used for immunoprecipitations of the H3.3 construct with interacting proteins from total protein extracts. Isogenic HEK293 cells were grown in DMEM media with 10% FBS in a Ø10 cm dish, harvested at subconfluency by cell scraping, rinsed twice with PBS and lyzed with 200 µl ice-cold lysis buffer containing 50 mM Tris-HCl (pH7.6), 150 mM NaCl, 1% NP-40, 0.5% sodium deoxycholate, and 0.1% SDS, supplemented with 2.5 mM MgCl_2_, 3 µl [25U/µl] Benzonase nuclease (Novagen, Millipore) and Complete protease inhibitors (Roche), Pepstatin A (Roche) and Aprotenin (Roche), and placed at +4 °C for 30 minutes on rotation. Cell debris was spun down and supernatant was diluted 1:2.5 in dilution buffer (10 mM Tris-HCl (pH7.5), 150 mM NaCl, 0.5 mM EDTA). Total protein concentrations were determined by BCA protein assays (Pierce, Thermo Scientific) and PAGE-gel followed by Coomassie (BioRad) staining to determine equal loading prior to immunoprecipitation. The equal loading-adjusted protein lysates were incubated with equilibrated 25 µl GFP-Trap-A bead slurry per manufacturers guidelines (Chromotek), washed and recovered with 2x Laemmli buffer (BioRad) supplemented with 10% ß-mercaptoethanol. A new PAGE-gel was run to separate the eluates, and stained with Silver stain kit (Pierce, Thermo Scientific) and Coomassie for verification. Each lane was divided into three equal portions and subjected to in gel tryptic digestion and LC-MS/MS separation with the Q Exactive Hybrid Quadrupole-Orbitrap Mass spectrometer instrumentation (Thermo Fisher Scientific). Protein scores were generated and analyzed by the SEQUEST algorithm (Thermo Fisher Scientific). We performed GO-term analysis using STRING (string-db.org). The heatmap of GO-terms were calculated based on z-score converted [−log_10_] *P*-value, scaled based on column direction.

### Splicing and Western blot validations

Purified total RNA (RNeasy kit, Qiagen) from 22 GCTB fresh frozen biopsies and 13 cell line samples (Supplementary Table S1) were used in the nCounter hybridization-based RNA transcript quantification technology (Nanostring). The validation set contained 12 samples not used in the test set. We had 39 genes designed for the nCounter gene expression custom codeset to validate the identified splicing defects and alternative starting sites seen in the six GCTB cell line sample set.

Western blot analysis was performed with standard PAGE and submarine protein transfer system (BioRad), and detection with the SNAP i.d. 2.0 protein detection system (Merck Millipore). Antibodies used were anti-GFP (ab290, Abcam), and anti-hnRNPA1L2 (ab180124, Abcam).

### Micrococcal nuclease assay

Chromatin from primary cells of GCTB was prepared according to a protocol from Soldi and Bonaldi, with slight modifications^45^. In brief, ~10^8^ cells from a Ø15 cm cell culture dish were washed with PBS and collected with a cell scraper. Cells were extracted in sucrose lysis buffer (15 mM HEPES pH7.5, 0.5 mM EGTA, 1 mM DTT, 15 mM NaCl, 60 mM KCl, 10% sucrose, 0.5% Triton X-100) and rotated for 10 minutes at 4 °C. The extract was put on a 20% sucrose cushion and centrifuged at 3200xRCF for 20 minutes at 4 °C. The pellet was washed with PBS and resuspended in 500 µl digestion buffer (50 mM Tris pH7.4, 0.32 M sucrose, 4 mM MgCl_2_, 1 mM CaCl_2_, and protease inhibitors). For every 20 µl nuclei suspension, 0.01 units micrococcal nuclease (MNase) was added and incubated at 37 °C for 0 (no enzyme), 10, 20, 40, 60 minutes as indicated in Fig. S5. Reaction was stopped by adding 2 µl 10 mM EDTA. RNA and protein were digested with RNaseA (Qiagen) and proteinase K (Invitrogen), respectively, prior to column purification with Qiaquick (Qiagen). Purified DNA were analyzed on a DNA12000 chip in BioAnalyzer (Agilent Technologies). Collected data was replotted in Excel (Microsoft).

### Bioinformatic analysis

For the microarray data, we applied the LIMMA package in Bioconductor for pre-processed normalization and filtering with detection *P*-value < 0.05^46^. We used linear modeling fitting and empirical Bayes smoothing method for statistical analysis to identify differential expression. Heatmaps and complete hierarchical clustering were generated with the heatmap3 package^47^. Gene set enrichment analysis (GSEA) was performed with the GSEA software (version 2.2.2 for microarray and version 2.2.3 for RNA-seq.)^48^.

For the RNA-sequencing data, we trimmed the reads using trimmomatic0.36^49^, mapped the reads with TopHat^50^ to the human genome build hg19, and assembled the reads with CuffLinks^51^. To calculate splicing events, we used the rMATS^52^ and SpliceR^53^ packages independently. rMATS computes the aberrant exon junctions from aligned reads using BAM to five different alternative splicing events categories (skipped exon, alternative 5′ splicing site, alternative 3′ splicing site, mutually exclusive exons, retained intron). SpliceR uses the cufflinks assembly to compute differences in transcripts in eight splicing categories (multiple exon skipping/inclusion, alternative transcription start site as well as five rMATS classes).

### Data availability

The transcriptome data from the two gene expression platforms have been deposited in the NCBI data gene expression omnibus (GEO) repository with the following accession numbers: Microarray (GSE102193) and RNA-seq. (GSE103559).

## Electronic supplementary material

Supplementary Figures

Supplementary Table S1

Supplementary Table S2

## References

[1] Merlo LM, Pepper JW, Reid BJ, Maley CC (2006). Cancer as an evolutionary and ecological process. Nat Rev Cancer.

[2] Morgan MA, Shilatifard A (2015). Chromatin signatures of cancer. Genes Dev.

[3] Plass C (2013). Mutations in regulators of the epigenome and their connections to global chromatin patterns in cancer. Nat Rev Genet.

[4] Schwartzentruber J (2012). Driver mutations in histone H3.3 and chromatin remodelling genes in paediatric glioblastoma. Nature.

[5] Wu G (2012). Somatic histone H3 alterations in pediatric diffuse intrinsic pontine gliomas and non-brainstem glioblastomas. Nat Genet.

[6] Lewis PW (2013). Inhibition of PRC2 activity by a gain-of-function H3 mutation found in pediatric glioblastoma. Science.

[7] Chan KM (2013). The histone H3.3K27M mutation in pediatric glioma reprograms H3K27 methylation and gene expression. Genes Dev.

[8] Bender S (2013). Reduced H3K27me3 and DNA hypomethylation are major drivers of gene expression in K27M mutant pediatric high-grade gliomas. Cancer Cell.

[9] Behjati S (2013). Distinct H3F3A and H3F3B driver mutations define chondroblastoma and giant cell tumor of bone. Nat Genet.

[10] Filipescu D, Szenker E, Almouzni G (2013). Developmental roles of histone H3 variants and their chaperones. Trends Genet.

[11] McKittrick E, Gafken PR, Ahmad K, Henikoff S (2004). Histone H3.3 is enriched in covalent modifications associated with active chromatin. Proc Natl Acad Sci USA.

[12] Wong LH (2009). Histone H3.3 incorporation provides a unique and functionally essential telomeric chromatin in embryonic stem cells. Genome Res.

[13] Szenker E, Ray-Gallet D, Almouzni G (2011). The double face of the histone variant H3.3. Cell Res.

[14] Elsaesser SJ, Goldberg AD, Allis CD (2010). New functions for an old variant: no substitute for histone H3.3. Curr Opin Genet Dev.

[15] Frey A, Listovsky T, Guilbaud G, Sarkies P, Sale JE (2014). Histone H3.3 is required to maintain replication fork progression after UV damage. Curr Biol.

[16] Huang C, Zhu B (2014). H3.3 turnover: a mechanism to poise chromatin for transcription, or a response to open chromatin?. Bioessays.

[17] Werner M (2006). Giant cell tumour of bone: morphological, biological and histogenetical aspects. Int Orthop.

[18] Fellenberg J (2013). Rescue of silenced UCHL1 and IGFBP4 expression suppresses clonogenicity of giant cell tumor-derived stromal cells. Cancer Lett.

[19] Liu L (2014). Enrichment of c-Met+ tumorigenic stromal cells of giant cell tumor of bone and targeting by cabozantinib. Cell Death Dis.

[20] Balke M (2013). Denosumab treatment of giant cell tumour of bone. Lancet Oncol.

[21] Boyle WJ, Simonet WS, Lacey DL (2003). Osteoclast differentiation and activation. Nature.

[22] Lacey DL (2012). Bench to bedside: elucidation of the OPG-RANK-RANKL pathway and the development of denosumab. Nat Rev Drug Discov.

[23] Zheng N, Fraenkel E, Pabo CO, Pavletich NP (1999). Structural basis of DNA recognition by the heterodimeric cell cycle transcription factor E2F-DP. Genes Dev.

[24] Osasan S (2016). Osteogenic Sarcoma: A 21st Century Review. Anticancer Res.

[25] Lewis PW, Elsaesser SJ, Noh KM, Stadler SC, Allis CD (2010). Daxx is an H3.3-specific histone chaperone and cooperates with ATRX in replication-independent chromatin assembly at telomeres. Proc Natl Acad Sci USA.

[26] Zhou X, Liao WJ, Liao JM, Liao P, Lu H (2015). Ribosomal proteins: functions beyond the ribosome. J Mol Cell Biol.

[27] Lin CJ, Conti M, Ramalho-Santos M (2013). Histone variant H3.3 maintains a decondensed chromatin state essential for mouse preimplantation development. Development.

[28] Dvinge H, Kim E, Abdel-Wahab O, Bradley RK (2016). RNA splicing factors as oncoproteins and tumour suppressors. Nat Rev Cancer.

[29] Jang CW, Shibata Y, Starmer J, Yee D, Magnuson T (2015). Histone H3.3 maintains genome integrity during mammalian development. Genes Dev.

[30] Ray-Gallet D (2011). Dynamics of histone H3 deposition *in vivo* reveal a nucleosome gap-filling mechanism for H3.3 to maintain chromatin integrity. Mol Cell.

[31] Hammond CM, Stromme CB, Huang H, Patel DJ, Groth A (2017). Histone chaperone networks shaping chromatin function. Nat Rev Mol Cell Biol.

[32] Buschbeck M, Hake SB (2017). Variants of core histones and their roles in cell fate decisions, development and cancer. Nat Rev Mol Cell Biol.

[33] Torres-Padilla ME, Bannister AJ, Hurd PJ, Kouzarides T, Zernicka-Goetz M (2006). Dynamic distribution of the replacement histone variant H3.3 in the mouse oocyte and preimplantation embryos. Int J Dev Biol.

[34] Santenard A (2010). Heterochromatin formation in the mouse embryo requires critical residues of the histone variant H3.3. Nat Cell Biol.

[35] Oristian DS (2009). Ribosomal protein L29/HIP deficiency delays osteogenesis and increases fragility of adult bone in mice. J Orthop Res.

[36] Lam YW, Lamond AI, Mann M, Andersen JS (2007). Analysis of nucleolar protein dynamics reveals the nuclear degradation of ribosomal proteins. Curr Biol.

[37] Matera AG, Wang Z (2014). A day in the life of the spliceosome. Nat Rev Mol Cell Biol.

[38] Chiou NT, Shankarling G, Lynch K (2013). W. hnRNP L and hnRNP A1 induce extended U1 snRNA interactions with an exon to repress spliceosome assembly. Mol Cell.

[39] Martincorena I, Campbell PJ (2015). Somatic mutation in cancer and normal cells. Science.

[40] Dawson MA (2017). The cancer epigenome: Concepts, challenges, and therapeutic opportunities. Science.

[41] Campanacci M, Baldini N, Boriani S, Sudanese A (1987). Giant-cell tumor of bone. J Bone Joint Surg Am.

[42] Enneking, W. F. A system of staging musculoskeletal neoplasms. *Clin Orthop Relat Res* 9–24 (1986). 3456859

[43] Chen HC (2005). Boyden chamber assay. Methods Mol Biol.

[44] Sander JD (2010). ZiFiT (Zinc Finger Targeter): an updated zinc finger engineering tool. Nucleic Acids Res.

[45] Soldi, M. & Bonaldi, T. The ChroP approach combines ChIP and mass spectrometry to dissect locus-specific proteomic landscapes of chromatin. *J Vis Exp*, 10.3791/51220 (2014). 10.3791/51220 PMC4166860 24747196

[46] Ritchie ME (2015). limma powers differential expression analyses for RNA-sequencing and microarray studies. Nucleic Acids Res.

[47] Zhao S, Guo Y, Sheng Q, Shyr Y (2014). Advanced heat map and clustering analysis using heatmap3. Biomed Res Int.

[48] Subramanian A (2005). Gene set enrichment analysis: a knowledge-based approach for interpreting genome-wide expression profiles. Proc Natl Acad Sci USA.

[49] Bolger AM, Lohse M, Usadel B (2014). Trimmomatic: a flexible trimmer for Illumina sequence data. Bioinformatics.

[50] Trapnell C, Pachter L, Salzberg SL (2009). TopHat: discovering splice junctions with RNA-Seq. Bioinformatics.

[51] Trapnell C (2010). Transcript assembly and quantification by RNA-Seq reveals unannotated transcripts and isoform switching during cell differentiation. Nat Biotechnol.

[52] Shen S (2014). rMATS: robust and flexible detection of differential alternative splicing from replicate RNA-Seq data. Proc Natl Acad Sci USA.

[53] Vitting-Seerup K, Porse BT, Sandelin A, Waage J (2014). spliceR: an R package for classification of alternative splicing and prediction of coding potential from RNA-seq data. BMC Bioinformatics.

