# Supplementary material for: The histone variant H3.3 G34W substitution in giant cell tumor of the bone link chromatin and RNA processing
Source: Sci Rep. 2017 Oct 18;7:13459. doi: 10.1038/s41598-017-13887-y (PMC5647428; doi:10.1038/s41598-017-13887-y)
Supplement: Supplementary file 1 — Supplementary Figures [file 41598_2017_13887_MOESM1_ESM.pdf]

Supplementary figures:

The histone variant H3.3 G34W substitution in giant cell tumor of the bone link chromatin and RNA processing

Jinyeong Lim[1], Joo Hyun Park[3], Annika Baude[2], Yeongran Yoo[1], Yeon Kyu Lee[3], Christopher R. Schmidt[2], Jong Bae Park[1], Jörg Fellenberg[4], Josef Zustin[5], Florian Haller[6], Irene Krücken[7], Hyun Guy Kang[1], Yoon Jung Park[3], Christoph Plass[2*], Anders M. Lindroth[1*]

Supplementary Figure 1

a

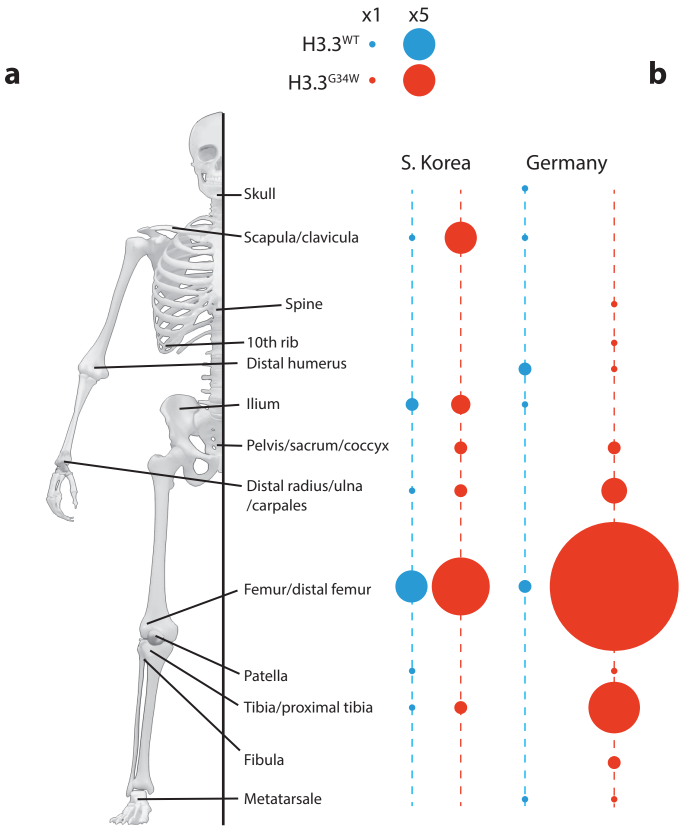

b

	S. Korea		Germany		
	WT	G34W	WT	G34W	All [%]
Scapula/Clavicle	1	5	1	0	8,4
Distal humerus	0	0	2	1	3,6
Distal radius/ulna/carpales	1	2	0	4	8,4
Skull	0	0	1	0	1,2
Rib	0	0	0	1	1,2
Spine	0	0	0	1	1,2
Pelvis/sacrum/coccyx	0	2	0	2	4,8
Ilium	2	3	1	0	7,2
Femur/distal femur	5	9	2	20	43,4
Tibia/proximal tibia	1	2	0	8	13,3
Fibula	0	0	0	2	2,4
Patella	1	0	0	1	2,4
Metatarsale/Talus	0	0	1	1	2,4
Sum	11	23	8	41	83

	H3.3G34W incidence [%]
Distal radius/ulna/carpales	86
Femur/distal femur	81
Tibia/proximal tibia	91

c

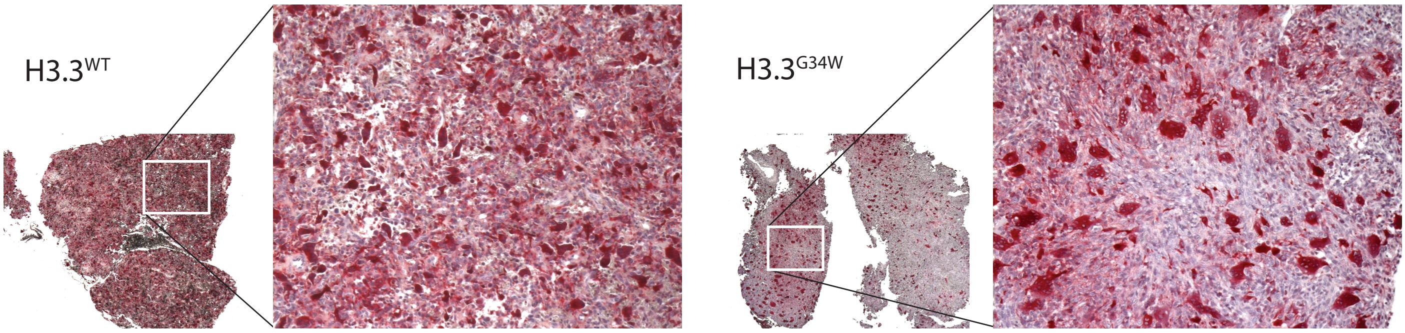

d

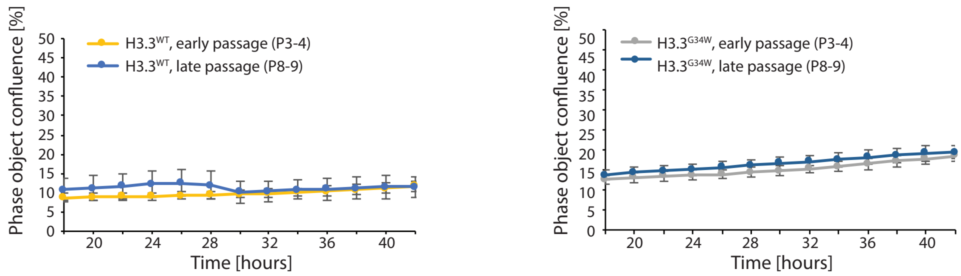

Fig. S1. Distribution of GCTB biopsies with $H3.3^{WT}$ or $H3.3^{G34W}$ in the long bones of the South Korean and German cohorts.

a) Schematic view of the distribution of the incidence of GCTB in the two cohorts throughout the human skeleton. Size of the circles indicate number of cases, $H3.3^{WT}$ (blue) and $H3.3^{G34W}$ (red). Illustration of the skeleton is an adjusted reproduction of an original by BruceBlaus as own work at the following link https://commons.wikimedia.org/w/index.php?curid=29849179. The original illustration is licensed under the Creative Commons Attribution 3.0 Unported license provided by the CC BY 3.0 (https://creativecommons.org/licenses/by/3.0/legalcode).

b) Number of biopsies at indicated anatomical locations, where green indicates no cases and red the most cases. Percentage of total irrespective of mutational status is listed in the rightmost white column. The percentage of $H3.3^{G34W}$ incidences at the most common GCTB prone areas of the long bones are indicated below the table as 86, 81, and 91%. Note that the number of samples are 83, where 3 samples are not included from the total because of lack of mutational status or location information and 2 samples are non-GCTB reference control cell lines. The total number of samples in this manuscript is as stated in materials and methods 88.

c) TRAP assay of GCTB biopsies, staining giant cells dark red (based on alkaline phosphatase) in one $H3.3^{WT}$ (left) and one $H3.3^{G34W}$ (right) sample. White square of the biopsy on the left indicate the position of the magnified picture on the right.

d) Experiment to determine if passage number influence proliferation measurement. IncuCyte confluency recordings based on $H3.3^{WT}$ (left panel) or $H3.3^{G34W}$ (right panel) in early (P3-4) or late (P8-9) passages, indicating that there are no significant differences between early and late passages in growth rate.

Supplementary Figure 2

a Biopsies and cell lines together

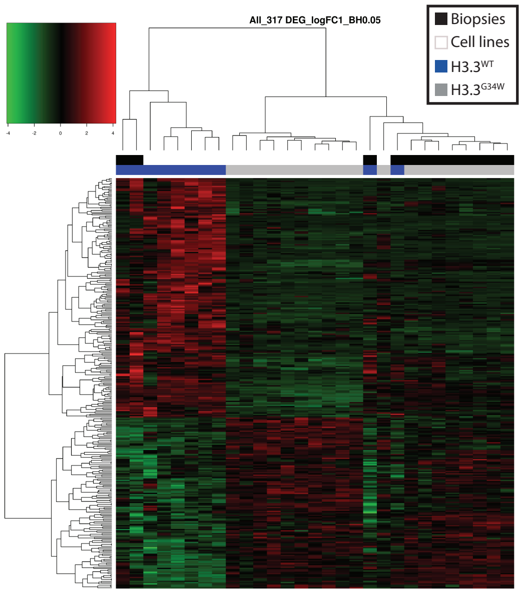

b

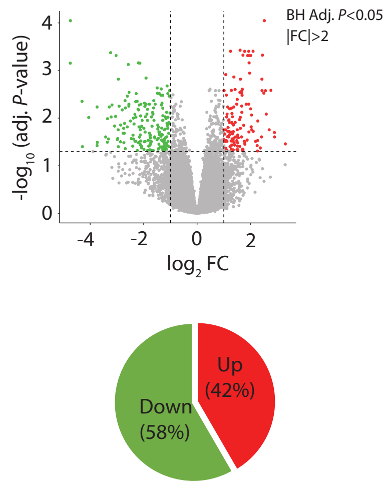

c Biopsies

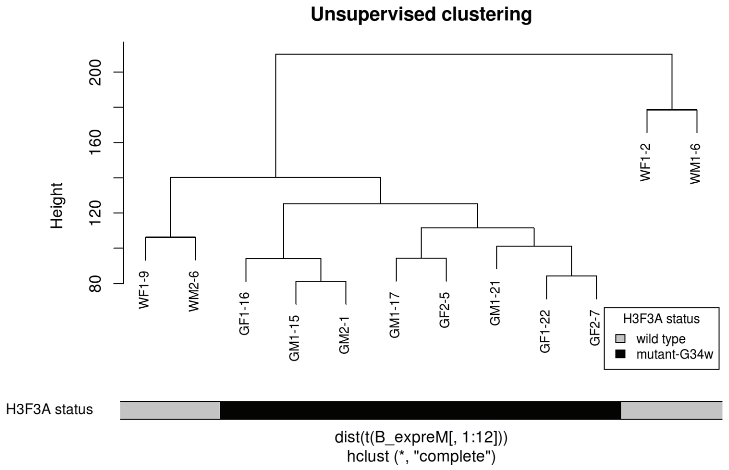

d Cell lines

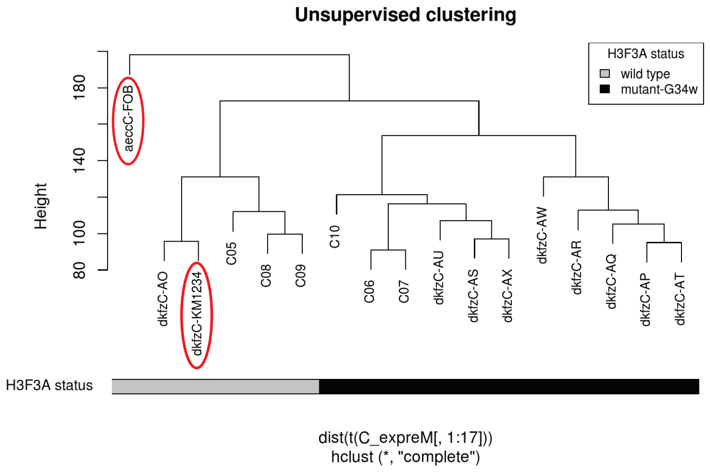

Fig. S2. Gene expression analysis from microarray of GCTB biopsies and established primary cell lines harboring $H3.3^{WT}$ or $H3.3^{G34W}$.

a) Heatmap with dendrograms of top 317 most differentially expressed genes in biopsies and primary cell lines in a combined analysis. $H3.3^{WT}$ (blue) and $H3.3^{G34W}$ (grey) samples cluster separately.

b) Volcano plot indicating that most genes are downregulated when comparing $H3.3^{WT}$ or $H3.3^{G34W}$. Fold change >2 indicated with red (up) and green (down) dots, where dots above line indicate adjusted *P*-value <0.05.

c) Dendrogram (unsupervised, complete clustering) of biopsies indicating that $H3.3^{WT}$ (grey) and $H3.3^{G34W}$ (black) cluster separately.

d) Dendrogram (unsupervised, complete clustering) of primary cell lines indicating that $H3.3^{WT}$ (grey) and $H3.3^{G34W}$ (black) cluster separately. The two non-GCTB cell lines hFOB1.19 (chondrosarcoma origin) and KM1234 (mesenchymal stromal cell origin) are indicated with red circles.

Supplementary Figure 3

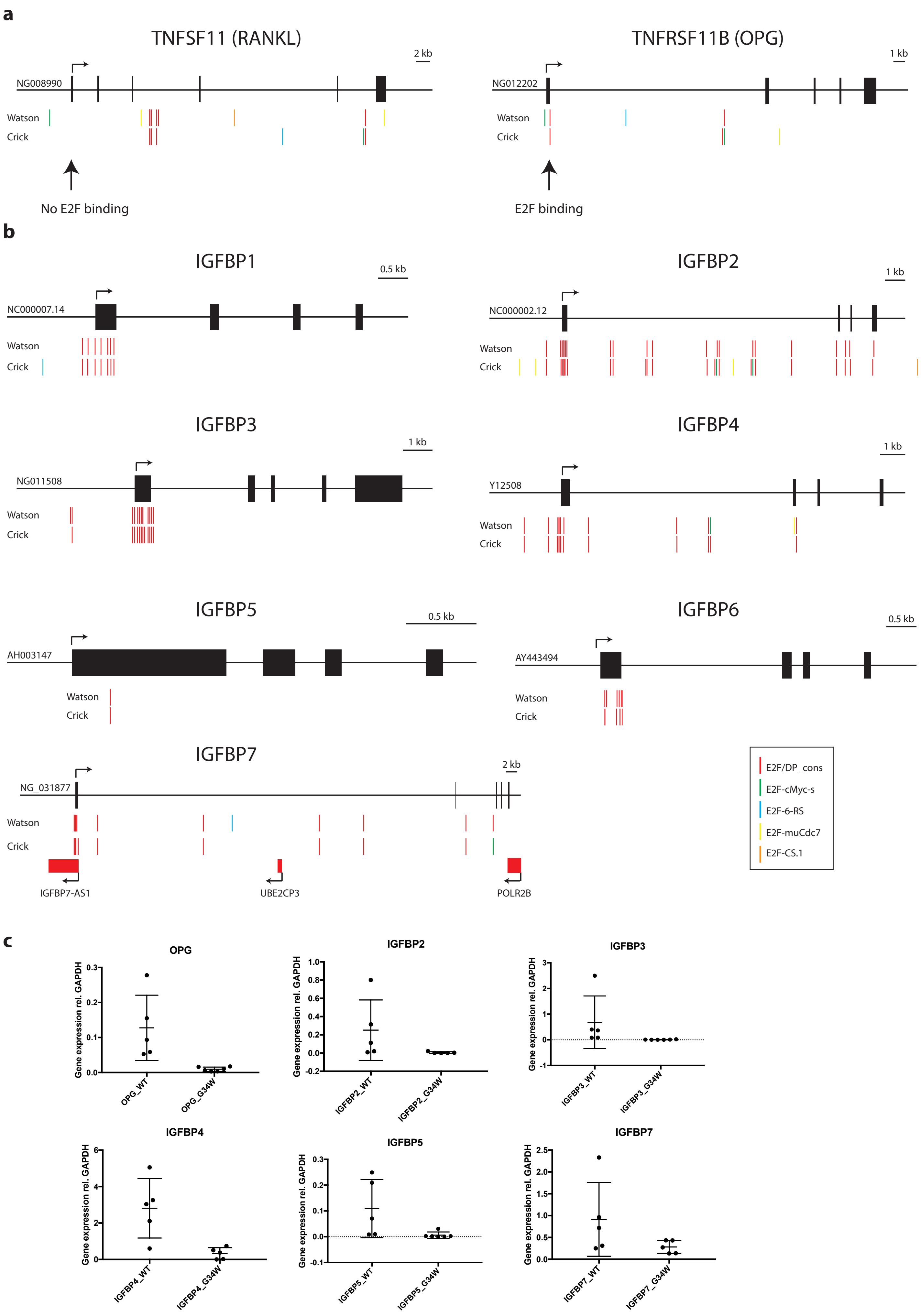

Fig. S3. E2F transcription factor targets of downregulated genes in GCTB with $H3.3^{G34W}$ compared to $H3.3^{WT}$.

a) RANKL and OPG are differentially targeted by E2F transcription factors, which is reflected by their gene expression pattern. OPG has E2F/DP (dimerizing protein or pocket proteins) at TSS but not RANKL.
b) The IGFBP family are targeted by E2F/DP at the TSS.
c) Plots showing RT-qPCR validations of the microarray analysis from Fig. 3 indicate that the trend is downregulation. A total of five $H3.3^{WT}$ and six $H3.3^{G34W}$ samples were used for the validation analysis.

Identification of the E2F target sequences were performed with the MacVector nucleic acid subsequence feature. References to binding: E2F/DP, seq.: SGCGCS (Genes & Development 13:666 -74(1999)); E2F_CS.1, seq.: TTTCGCGC (EMBO J 6: 2061-8 (1987)); E2F-c-Myc-s, seq.: GATCGCGC (Proc Natl Acad Sci U S A 86: 3594-3598 (1989)); E2F-6_RS, seq.: TTTCCCGC (Oncogene 17: 611-23 (1998)); E2F-muCdc7, seq.: GCCAAAATTC (J Biol Chem 273: 23248-23257 (1998)).

Supplementary Figure 4

a

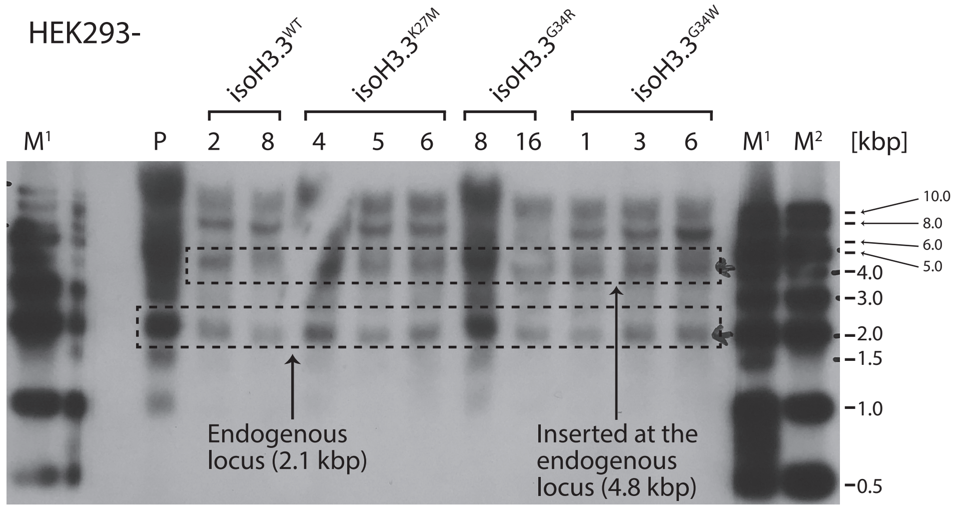

b

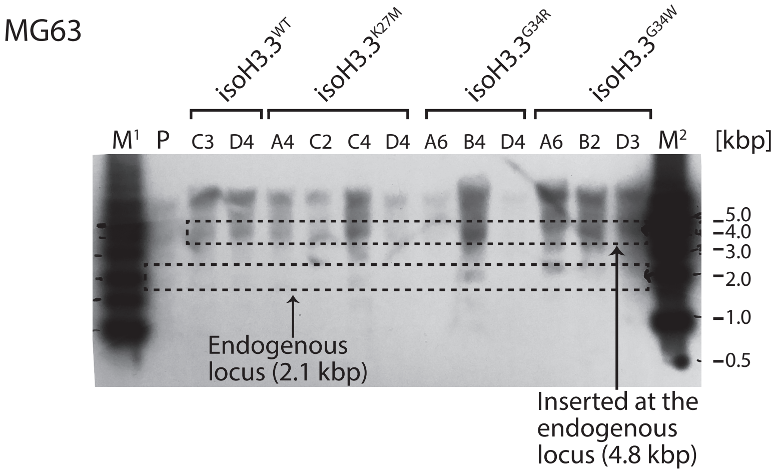

M[1]: 1kb ladder Plus (ELPIS BIOTECH)

M[2]: 1kb ladder (ELPIS BIOTECH)

P: parental cell lines prior to transgenics (HEK293 without T-antigen, or MG63)

2, 8, 4, etc.: indicate individual isogenic clones in HEK293

C3, C4, A4, etc.: indicate individual isogenic clones in MG63

c

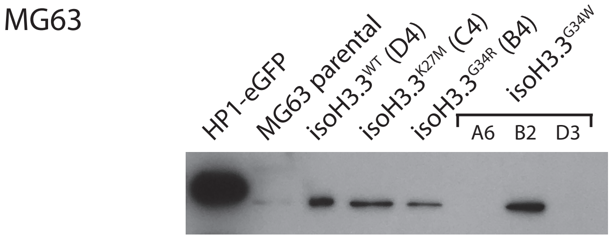

d

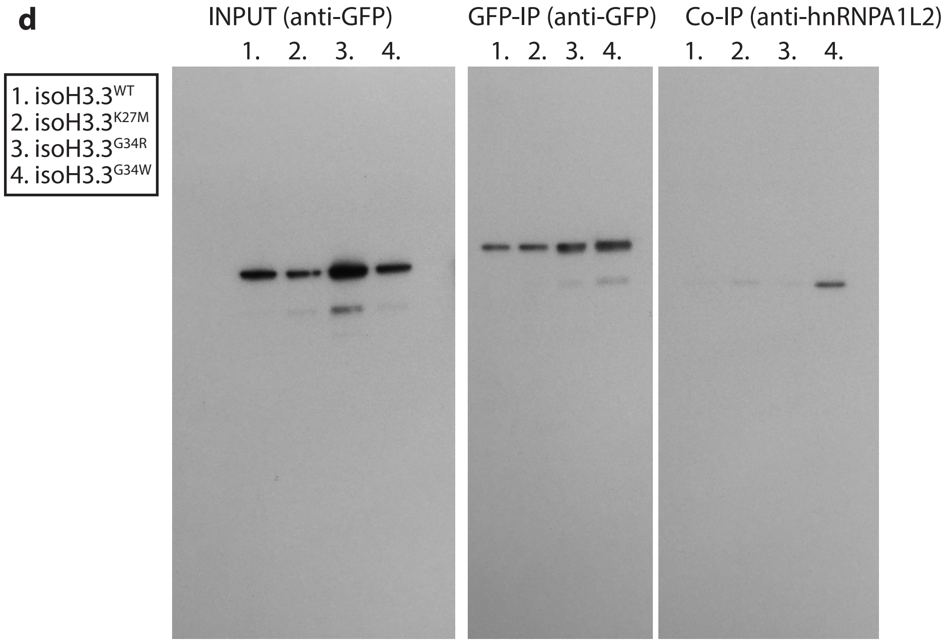

Fig. S4. Verification of correct zinc finger gene targeting to the endogenous H3F3A locus and GFP expression.

a) Ten gene targeting clones in HEK293 (without T antigen) cells were subject to Southern blot analysis with a probe binding over a targeting construct-specific *Bgl*II-cut site, producing a 4.8 kb fragment from the correctly targeted locus and a 2.1 kb fragment from the non-targeted, endogenous locus.
b) Same as A) but correct targeting cell verification in the osteosarcoma cell line MG63.
c) Western blot analysis for GFP expression emanating from the isogenic GFP-expressing constructs described in B).
d) Original uncropped Western blot analysis of GFP-IP shown in Fig. 3i with hnRNPA1L2 detection in the right panel.

Supplementary Figure 5

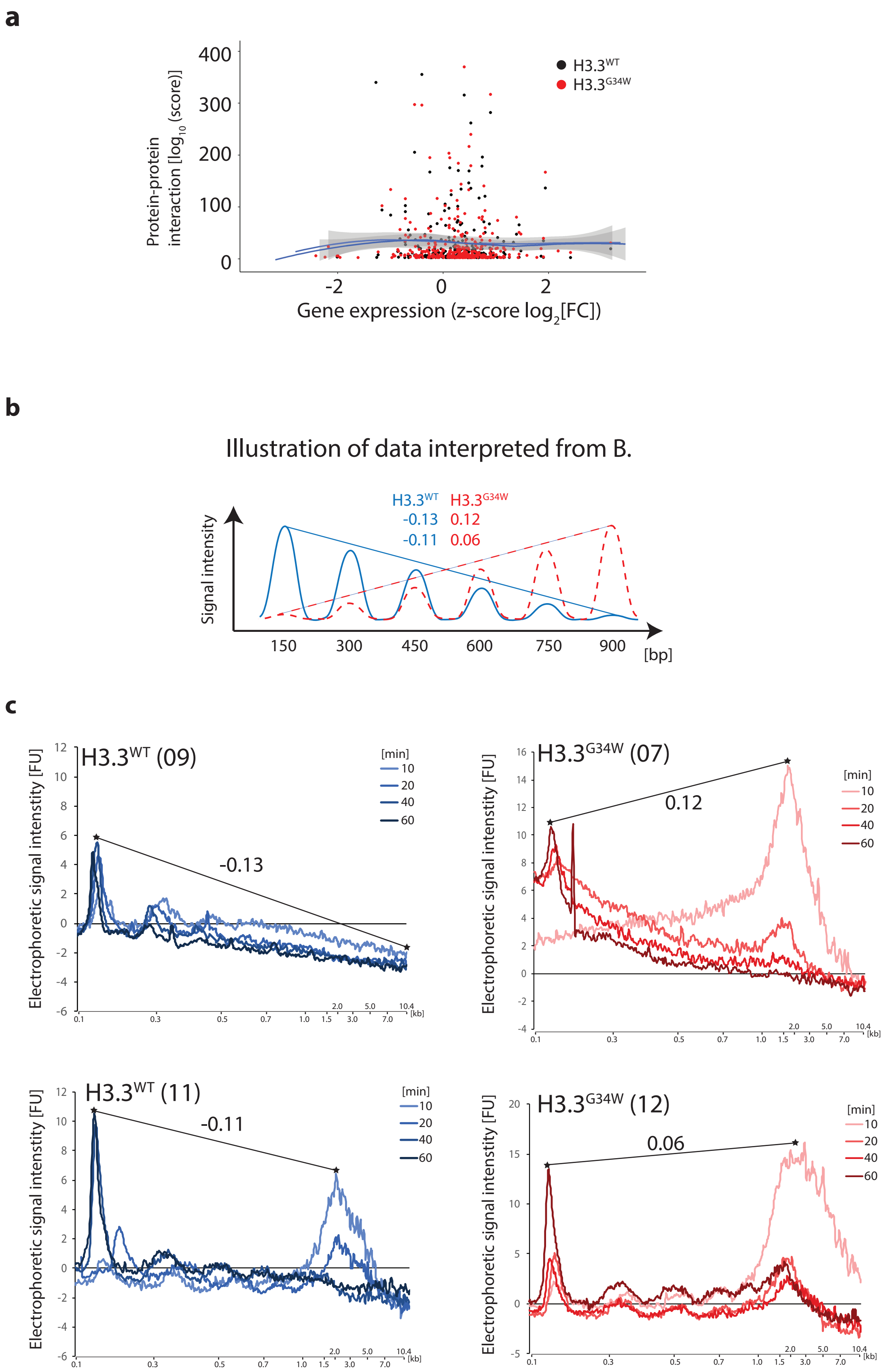

Fig. S5. Gene expression and co-immunoprecipitation protein interaction lack correlation. Micrococcal nuclease (MNase) assay indicating elevated chromatin compaction in H3.3^{G34W} compared to H3.3^{WT}.

a) Scatter plot of protein-protein interaction score of identified proteins against their respective gene expression level (z-score, log_2 fold change). The plot indicates that there is no correlation between low or high interaction scores and the corresponding gene expressions (linear regression H3.3^{WT} r^2=0.00072, H3.3^{G34W} r^2=0.00072).

b) Illustration of data interpretation from electropherograms in Fig. 4A and Fig. S5C) indicating that nucleosome-size pieces of DNA is enriched in the mono- to oligonucleosome range in H3.3^{WT} (blue), compared to the enrichment of polynucleosomes in H3.3^{G34W}. Line from left to right has been drawn between highest peak of undigested to digested chromatin to calculate a slope that would indicate level of chromatin compaction. Consequently, an open chromatin produces a negative slope, while a closed chromatin produces a positive slope.

c) MNase assay followed by separation in BioAnalyzer (Agilent) and electropherogram data in fluorescence units [FU] indicating digestion of DNA into mononucleosomes (far left, 150 bp) or polynucleosomes (far right, >1kb). Lines indicate how slopes have been calculated between the two extremes (mononucleosomes to polynucleosomes). MNase assay from different time points of incubation with MNase (0 (no enzyme), 10, 20, 40, 60 minutes). Slope based on Δfluorescence intensity[peak^{highest}-peak^{lowest}]/Δsize [bp peak^{highest}-peak^{lowest}].

Supplementary Figure 6

a Alternative start

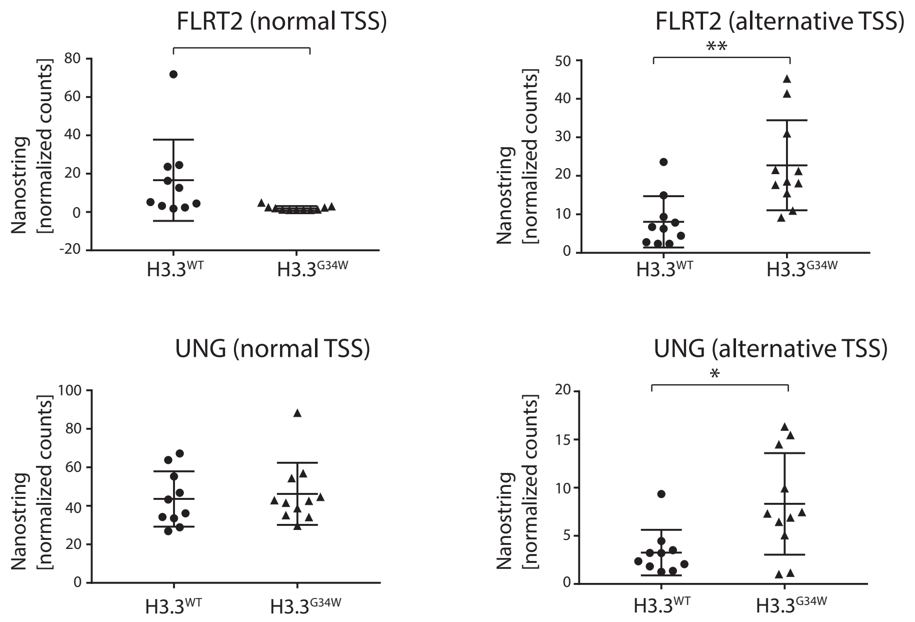

b Exon inclusion

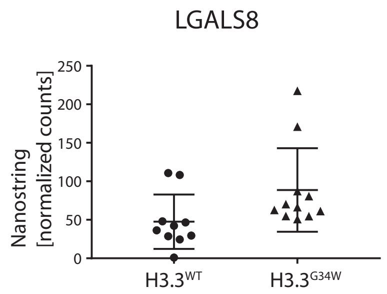

c Exon skipping

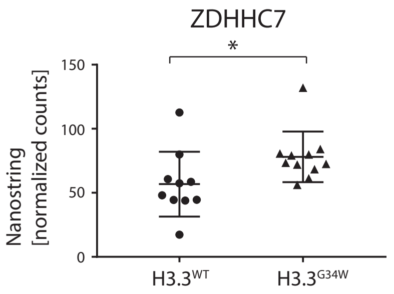

Fig. S6. Elevated alternative start and splicing aberrations in H3.3^{G34W} compared to H3.3^{WT}.

a) FLRT2 and UNG are two genes that show alternative start sites in H3.3^{G34W} downstream of the first exon (altTSS). This is accompanied by a decrease of the canonical first exon (TSS), especially prominent in FLRT2.

b) The LGALS8 (galectin) locus shows increased exon inclusion. Galectins are commonly expressed in tumor tissue and may be involved in splicing itself.

c) The ZDHHC7 (zinc finger DHHC-type containing 7) locus shows increased exon skipping. The zinc finger ZDHHC7 mediates palmitoylation which regulates cell polarity and tumor suppression.
